# I Rise to Explain

**Published:** 1877-04

**Authors:** C. E. Case

**Affiliations:** San Francisco


### I RISE TO EXPLAIN.

BY C. E. CASE, D. D. S., SAN FRANCISCO.

In the February number of the REGISTER there is an editorial entitled "Celluloid—what about it?" in which Dr. Chase is quoted as saying that celluloid has proven a failure in his hands, and he has been advising young dentists to submit to Josiah's demands and work rubber. Now for the honor of the profession I hope he will cease giving such advice, and try celluloid again, and as the editor of the REGISTER calls on some one to "rise and explain," I take the opportunity thus offered to do so.

I will give my experience, and also my process, and feel confident that if all dentists would work celluloid as I do they would have no more trouble than I have, and that is none whatever.

There are three principal causes of failure in the working of the material. There is no cause for failure in the material itself. I have found after patient trials that there is no apparatus, which has as yet been brought before the profession, that will at all compare, in the uniform perfection of it's results, with the old original oil apparatus, using glycerine instead of oil. Here we have the first and most common cause of failure. Dry heat (hot air) is not what is wanted. The material will not work freely and well in air highly heated. Steam is a failure, as we are almost certain to either get the heat too high, or else by testing the safety valve often we allow the steam to escape, but fail to reduce the temperature and are thus deceived by the thermometer. The second cause of failure is lack of cleanliness in the apparatus and the various stages of the manipulation. Many dentists work rubber in a very slovenly manner and expect celluloid to be perfect under the same dirty treatment. They will find it is liable to fail unless it be kept perfectly clean all the time it is going through the moulds. Third and lastly, the flasking of a plate of celluloid is different from that of rubber. Celluloid does not flow like mush under pressure, but there must be considerable care and skill exercised in the flasking and cutting of gateways for the excess of the material. Before flasking it will pay to stop for a few minutes and reflect upon the character, shape of mouth and gums, position of teeth, etc., and determine just where we want the material to flow and where not. Always flask (except where no gum is needed) so that the teeth will separate from the model. Cut the gateways at the points where you intend to make gums and no place else. Separate the flask in the usual way and oil both sides of it thoroughly. After cutting the plate (if for a partial set) as near as you think proper to meet the requirements of the case place it in water and boil it. Then while it is still hot and soft place it upon the model and by pressure with the fingers shape it as near as you can to fit the model. Now place the whole flask in the clamp and the clamp in the tank of glycerine, close the clamp so that there is just enough pressure to hold the plate in place. Heat up until the glycerine boils. Let the thermometer be laid aside. You do not need it, for you can not heat the glycerine above it's boiling point, and that temperature is just right to mould celluloid successfully. When the glycerine boils, commence and turn slowly and gently with the thumb and fingers of one hand, stopping at intervals, as the resistance becomes greater. In this way the flask can be very nearly closed. Then cease turning for about five minutes, in order to allow the material to accommodate itself to the shape of the mould, then commence again and by increasing force, screw the clamp down firmly until the flask is closed, wait five minutes and then immerse in cold water or let it cool off in the tank, but let it be perfectly cold before it is opened.

By following this method I have made nearly three hundred plates in the last two years, and have yet to see a single one of them that has either warped or shrank from the teeth been fractured or changed its color, if properly cleansed and cared for. If people who use artificial teeth were as particular in keeping their mouths clean as they are with their hands and faces, we would hear no complaints about celluloid changing color. I get a perfect fit every time. My patients say that it is far more pleasant in the mouth than rubber, in fact I could mention quite a number of cases in which I have replaced rubber with celluloid with the happiest results, the inflamed condition of the mouth rapidly subsiding and the heated sensation passing away.

Now to sum up my experience I find as follows: Celluloid is the toughest and strongest material that has as yet been introduced to the profession; it is the lightest; best colored; it is the best of all materials for cheap bases. Using it for attachment upon gold plates I consider it next to continuous gum. If worked with care, cleanliness, the proper apparatus and plenty of time, it is the easiest worked and most satisfactory in every respect of any material I have as yet heard of. I am of the opinion that where failures have been made it was the fault of the operator and not of the material. Steam, hot air, etc., are failures, and the most beautiful results can only be obtained with glycerine. If my plan is followed in every particular I am sure that Josiah will have no terrors for the profession, and his pet rubber will take a back seat.

Celluloid is the *ne plus ultra* of the cheap bases.

